# Optimization of Veterinary Antimicrobial Treatment in Companion and Food Animals

**DOI:** 10.3390/antibiotics11081137

**Published:** 2022-08-22

**Authors:** Nikola Puvača

**Affiliations:** 1 Department of Engineering Management in Biotechnology, Faculty of Economics and Engineering Management in Novi Sad, University Business Academy in Novi Sad, Cvećarska 2, 21000 Novi Sad, Serbia; nikola.puvaca@fimek.edu.rs; 2 Faculty of Biomedical and Health Sciences, Jaume I University, Avinguda de Vicent Sos Baynat, s/n, 12071 Castelló de la Plana, Spain

Several international strategies for antimicrobial stewardship have been developed in response to the global crisis of antimicrobial resistance [1]. The truth of the matter is that despite all of their good intentions, these broad strategies are only slowly being implemented in the real world. Humans and animals are affected by antibiotic-resistant bacteria, which means that to combat this problem, both sectors must be considered. An important contributing factor to antimicrobial resistance is the use of antimicrobials [2]. It is undeniable that the use of antibiotics in companion and food animals, as well as antimicrobials, is beneficial in preventing diseases and improving their performance and overall welfare [3,4].

A shortage of experts in key disciplines related to antimicrobial stewardship in veterinary medicine makes it difficult to develop antimicrobial treatment guidelines. Diagnostic tests are also inferior to those in human microbiology and do not provide sufficient information, making it difficult to identify who is using antimicrobial products and when [5]. In both companion and food animals, antimicrobial resistance monitoring has posed several problems, including the use of appropriate methods for gathering data at the level of the animal, as well as the choice of metrics for measuring antimicrobial resistance and the health of animal populations at risk [6].

A Special Issue was devoted to researchers interested in monitoring antimicrobial resistance in animals, and optimizing antimicrobial use in veterinary medicine with a special focus on improving antimicrobial treatment guidelines, diagnostic procedures, and antimicrobial stewardship in companion and food animals.

As a very successful endeavor, this Special Issue has gathered more than 30 papers, of which a total of 17 high-quality reviews and research articles were positively scored, accepted, and published [7].

This Special Issue has welcomed submissions from a very diverse range scientific themes, from the antibiotic susceptibility of *Staphylococcus* species isolated in raw chicken meat from retail stores [8] to comparative evaluations of *qnrA*, *qnrB*, and *qnrS* genes in *Enterobacteriaceae* ciprofloxacin-resistant cases in swines [9]. Roque-Borda et al. [10] have investigated a Ctx(Ile^21^)-Ha antimicrobial peptide, evaluating the effects of its application in laying chicks challenged with *Salmonella* Enteritidis, resistant to nalidixic acid and spectinomycin, and achieving very promising results. Previously, natural alternatives to antibiotics usage in the form of essential oils was investigated in vitro [11], as well as in vivo, in cattle [12].

Xu et al. [13] conducted a study that aimed to examine the pharmacokinetics of doxycycline in yellow catfish (*Pelteobagrus fulvidraco*) and to calculate related pharmacokinetic-pharmacodynamic parameters of doxycycline against *Edwardsiella ictaluri*. The obtained results of this fascinating study suggest that administration of doxycycline at 20 mg/kg every 96 h is a preferable regimen in yellow catfish. Furthermore, Little et al. [14] revealed that pigs reared on many farms are mass-medicated for short periods with antibiotics through their drinking water to control bacterial pathogen loads and, if a disease outbreak occurs, to treat pigs until clinical signs are eliminated. Based on that fact, a survey was conducted including managers of 25 medium-to-large single-site and multi-site pig farming enterprises across eastern and southern Australia using a mixed methods approach. Little et al. [14] found wide variation in the antibiotics administered, the choice and use of dosing equipment, the methods for performing dosing calculations and preparing antibiotic stock solutions, the commencement time and duration of each daily dosing event, and the frequency of administration of metaphylaxis. This interesting and practical research has revealed that farm managers need to be provided with measurement systems, technical guidelines, and training programs to improve their stewardship. 

Several articles have revealed interesting and promising results of antibiotics usage and the prevalence of extended-spectrum cephalosporin-resistant *Enterobacterales* in horses, donkeys [15], pigs, chickens, fish [16], dogs [17], and cats [18]. When it comes to diseases in ruminants, a pilot study conducted in Sweden has presented the effects of benzylpenicillin, oxytetracycline, and florfenicol in the treatment of acute undifferentiated respiratory disease [19]. Furthermore, results of Petrocchi-Rilo et al.’s [20] investigation have confirmed that antimicrobial resistance genes in porcine *Pasteurella multocida* are not associated with its antimicrobial susceptibility pattern.

Another very interesting research with the application of antibiotics through drinking water of lambs has been presented by Ferran et al. [21]. Based on the obtained results, delivery by drinking water resulted in a very high interindividual variability of sulfadimethoxine plasma concentrations, meaning that although disease spread could be controlled at the group level, some individuals would inevitably be under- or over-exposed to the antibiotic.

Vilaró et al. [22] investigated the antimicrobial susceptibility patterns of swine respiratory pathogens in Spain from 2017 to 2019, while Mileva et al. [23] presented their results of oxytetracycline pharmacokinetics after intramuscular administration in cows with clinical metritis associated with *Trueperella pyogenes* infection in Bulgaria.

Puvača and de Llanos Frutos [24] presented evidence that antimicrobial resistance has become a big problem in recent years on a global scale. Public healthcare systems all over the world are faced with a great challenge in this respect. Many bacteria can cause infections in humans and animals alike, but somehow, it seems that the greatest threat nowadays comes from the *Enterobacteriaceae* members, especially *Escherichia coli*. Puvača and de Llanos Frutos [24] have stated that it is evident that the epidemiology of *E. coli* strains and resistance mechanisms that they have developed over time are extremely significant topics, and all scientific findings in this area will be of vital importance in the fight against infections caused by these bacteria.
